# Development and validation of a pretreatment prognostic index to predict death and lung metastases in extremity osteosarcoma

**DOI:** 10.18632/oncotarget.5276

**Published:** 2015-10-01

**Authors:** Bo Wang, Jian Tu, Junqiang Yin, Changye Zou, Jin Wang, Gang Huang, Xianbiao Xie, Jingnan Shen

**Affiliations:** ^1^ Musculoskeletal Oncology Center, the First Affiliated Hospital of Sun Yat-Sen University, Guangzhou, Guangdong, China

**Keywords:** osteosarcoma, survival outcomes, lung metastases, pretreatment prognostic index

## Abstract

**Background:**

To develop a prognostic index to predict the 5-year overall survival (OS) and 5-year lung metastasis-free survival (LMFS) of patients with extremity osteosarcoma at the time of diagnosis.

**Methods:**

We retrospectively evaluated 454 patients with extremity osteosarcoma at our center from 2005 to 2013. The cohort was randomly divided into training and validation sets. The association of potential risk factors with OS and LMFS was assessed by Cox proportional hazards analysis in the training set, and a prognostic index was created according to scores that were proportional to a regression coefficient for each factor. This prognostic index was assessed in the validation set.

**Results:**

For the 5-year OS, 5 independent prognostic factors were identified: tumor size, Enneking stage, pretreatment platelet, alkaline phosphatase(ALP), and neutrophils. The multivariate Cox model identified tumor size, pretreatment platelets, ALP, and neutrophils as associated with the 5-year LMFS. A prognostic index for death and lung metastases was calculated. Three risk groups were defined for each survival point: low, intermediate, and high risk for the 5-year OS; low, intermediate, and high risk for the 5-year LMFS. The C statistic for the 5-year OS was 0.723 in the training set and 0.710 in the validation set. The C statistic for the 5-year LMFS was 0.661 and 0.693 respectively.

**Conclusion:**

This prognostic index is based on routine tests and characteristics of extremity osteosarcoma patients and is a useful predictor of OS and lung metastases. This index could be applied to clinical practice and trials for individualized risk-adapted therapies.

## INTRODUCTION

Osteosarcoma is the most common malignant bone tumor in children and adolescents, although it accounts for less than 0.5% of all cancers [1]. With the addition of neo-adjuvant chemotherapy and adjuvant chemotherapy, the rates of overall survival (OS) and salvage surgery have increased over the last three decades. For patients with non-metastatic osteosarcoma, 5-year OS has increased to 60 to 70%, but this rate decreases to 20% when metastases occur [2]. Despite treatment improvements, marked heterogeneity in patient survival is observed.

Currently, the prognosis of osteosarcoma patients is primarily evaluated based on the Enneking staging system. A discrepancy often occurs between clinical outcomes and Enneking stage. Additionally, a limited number of clinico-pathological factors are available to predict the outcomes of osteosarcoma patients, especially at the time of diagnosis, which has made early identification and risk stratification therapy difficult in clinical practice. To the best of our knowledge, pretreatment serum alkaline phosphatase (ALP) levels are widely accepted as a predictor of osteosarcoma patient survival. Our previous study also demonstrated the predictive value of ALP levels on the OS and disease-free survival (DFS) of osteosarcoma patients [3]. However, osteosarcoma has a remarkably heterogeneous clinical behavior, and no single factor can accurately predict its prognosis. The need to develop effective markers to predict the OS and risk of lung metastases of osteosarcoma patients and to develop individualized treatments is great.

Although cancer outcomes depend on the genetic basis of the cancer, increasing evidence suggests that systematic inflammatory responses and coagulation are associated with survival outcomes in patients with different tumor types [4–6]. Additionally, the systematic inflammatory response and coagulation can be assessed by measurements of routine blood test markers, such as albumin, white blood cells (WBCs), neutrophils, lymphocytes and platelets (PLTs). Moreover, a growing body of evidence indicates that several systemic inflammatory factors are related to the survival of soft tissue sarcoma patients [7–10]. Therefore, continuing efforts to investigate prognostic indexes related to the survival of osteosarcoma patients are needed.

In this study, we aimed to evaluate the relationship between clinical factors and survival outcomes and to develop a prognostic scoring system for osteosarcoma patients that could be used at the time of diagnosis.

## MATERIALS AND METHODS

### Patients

Patients with newly confirmed osteosarcoma who underwent standard chemotherapy and surgery between January 2005 and December 2013 at the First Affiliated Hospital of Sun Yat-sen University were retrospectively enrolled. Then, 75% of the included patients were randomly selected as the training set to explore the predictive value of clinical factors from routine blood tests and to develop a prognostic index. The remaining 25% of the included patients were selected as the validation set to assess the prognostic score index. The Institutional Ethical Board of our hospital approved this study.

The criteria for case inclusion were as follows: (1) histologically confirmed osteosarcoma by needle biopsy; (2) no prior chemotherapy or radiotherapy prior to diagnosis; (3) receipt of first-line neo-adjuvant chemotherapy, operation and adjuvant chemotherapy; (4) receipt of at least 1 cycle of neo-adjuvant chemotherapy and 3 cycles of adjuvant chemotherapy; and (5) presence of a tumor located in an extremity. Patients with evidence of infectious or inflammatory diseases were excluded. Patients with symptoms such as fever or cough or who exhibited urethral stimulation symptoms or signs such as lung rales were excluded because these symptoms were considered evidence of potentially infectious or inflammatory diseases. Then, procalcitonin and C-reactive protein assessments, blood culture, urine culture and lung X-ray were performed according to the patient's signs and symptoms. If any positive results were obtained, the patient was excluded following a discussion. Patients with a Karnofsky Performance State score <70 were excluded [11]. 15 Patients without completed follow-up information were excluded.

### Treatment

**Figure 1:**
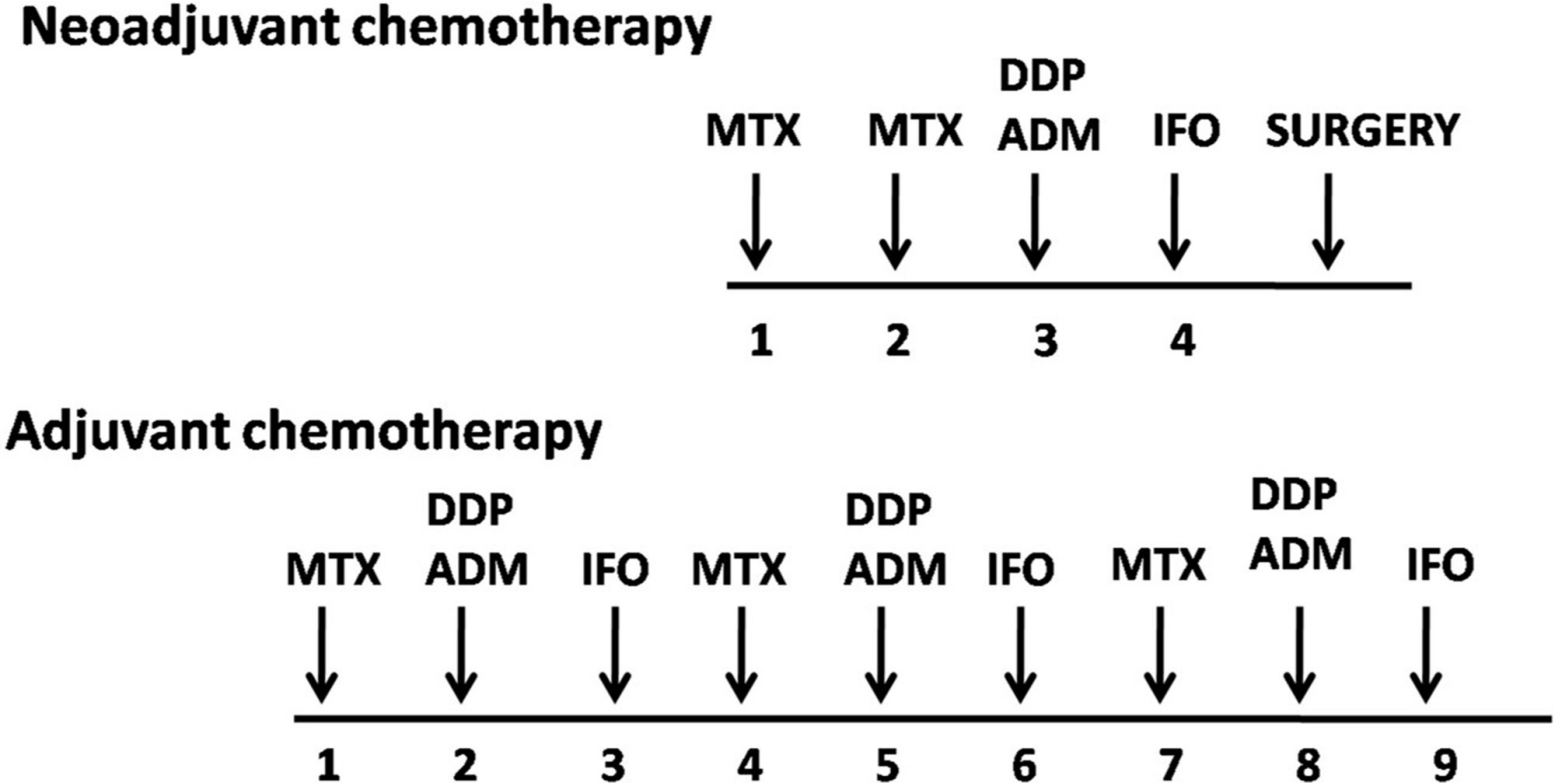
The chemotherapeutic agents and the treatment protocol of 454 patients

For definitive Enneking staging, a needle biopsy was performed for each patient. A computed tomography scan of the lung and a whole-body emission computed tomography scan were also performed. A total of four commonly used chemotherapeutic agents were administered according to the scheme presented in Figure 1: methotrexate, cisplatin, doxorubicin, and ifosfamide [3]. The interval between rounds of chemotherapy was 2 to 3 weeks. Then, 2 or 3 weeks after surgery, the patients would receive adjuvant chemotherapy if no complications were noted. All patients received standard chemotherapy including Methotrexate with different dose. For those >30 years old, 8–10 g/m2 of methotrexate was administered to reach a peak blood concentration of over 800 umol/L. and for those younger patients, 10–12 g/m^2^ of methotrexate was administered to reach a peak blood concentration of over 1000 umol/L, and according to literatures, both dose were proved to be effective [12, 13].

### Methods

Blood samples were obtained before the initial neo-adjuvant chemotherapy to measure the levels of albumin, WBCs, neutrophils, lymphocytes, PLTs, fibrinogen degradation products (FBGs), lactate dehydrogenase (LDH) and ALP. Because age influences ALP expression [14], 150 U/L was regarded as the upper serum ALP limit in patients less than 18 years, while 110 U/L was considered the limit in those 18 years and older.

### Statistical analyses

SPSS (version 19.0, Chicago, IL, USA) statistical software and R (version 3.0.1) were used for statistical analysis. Categorical variables were expressed as numbers and percentages, and the χ^2^ test was used to compare differences between groups. Continuous variables were presented as means and standard deviations, and means were compared using the *t*-test. OS was defined as the time from diagnosis to death from any cause or to the last follow-up visit. Lung metastasis-free survival (LMFS) was defined as the time from diagnosis to the detection of lung metastasis. Surviving patients were censored in the analysis of OS. Patients with lung metastasis at the time of diagnosis were censored in the analysis of LMFS.

Kaplan–Meier survival analysis was performed to estimate OS and LMFS, and the log rank test was used to compare rates between two groups. Univariate and multivariate analyses were performed for prognostic factors using the Cox proportional hazard model. Variables identified as significant by univariate analysis were selected to test with the Cox proportional hazard model. A *P*-value ≤ 0.05 was considered indicative of a significant difference.

The prognostic index was formulated based on Cox proportional hazards analysis, which has been used in many previous studies to develop prognostic systems, such as the system reported by Rassi [15]. The prognostic score was assigned based on risk factors that were identified by multivariate analysis and weighted points proportional to the β-regression coefficient values, which were based on the linear transformation (rounded to the nearest integer). The coefficient of each variable was divided by the lowest β value and rounded to the nearest integer. The prognostic factor with the lowest β-regression coefficient was assigned a prognostic score of 1. Thereby, a prognostic score was calculated for each patient. Patients in the training set were divided into different subgroups based on their prognostic index value. Kaplan–Meier survival analysis was performed, and different subgroups were compared; subgroups without a significant difference in 5-year OS or LMFS were combined to form three groups [16]. Then, the patients in the training and validation sets were divided into three groups: patients at low risk, patients at intermediate risk, and patients at high risk for 5-year OS and lung metastases. The C statistic was used to assess the predictive accuracy of the prognostic scoring system between the training and validation sets. The C statistic was calculated as (P_high_–P_low_) / 100, where P_high_ was the probability of death predicted for a patient in the group with the worst prognosis and P_low_ was the probability of death predicted for a patient in the group with the best prognosis [17].

## RESULTS

### Clinico-pathological patient characteristics

**Figure 2:**
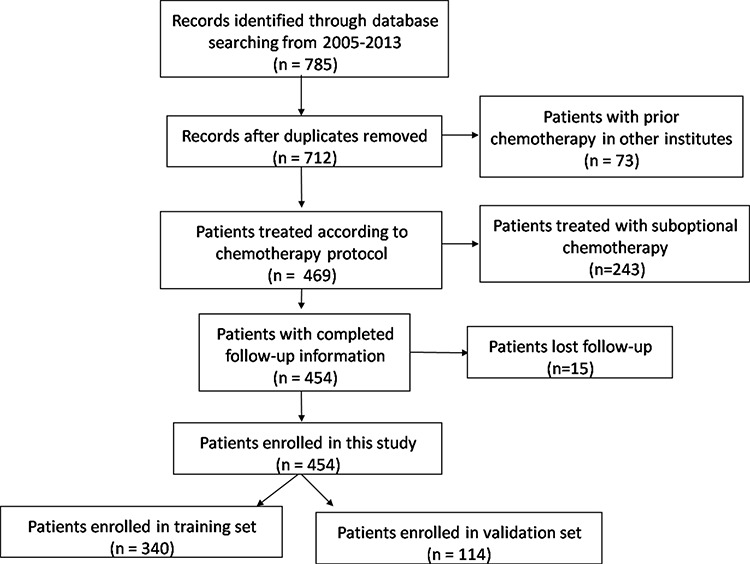
Flow chart for patients selection in this study

**Figure 3:**
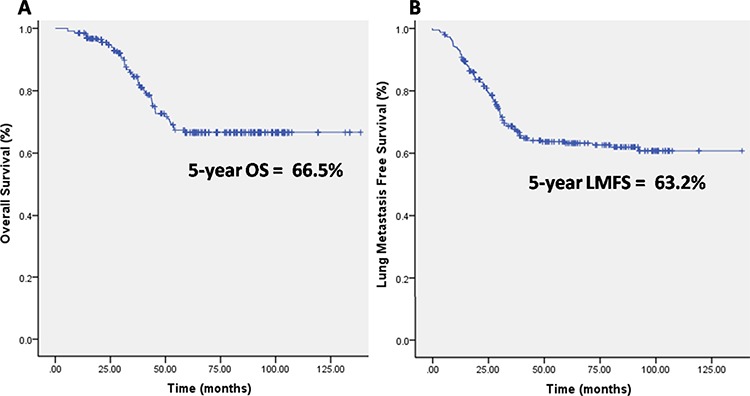
**A.** Kaplan-Meier curves showing the overall survival (OS) of the included patients. **B.** Kaplan-Meier curves showing the lung metastasis free survival (LMFS) of the included patients.

A total of 454 patients were included in this retrospective study (Figure 2). In total, 340 patients were included in the training set, and 114 patients were included in the validation set (Table 1). The mean and range of the age of the included patients were 17.2 and 6–55, respectively. The mean and range of the age of the patients in the training set were 17.2 and 6–53, respectively. The mean and range of the age of the patients in the validation set were 17.1 and 7–55, respectively. There were only 10 (2.2%) patients >40 years of age. Six of these patients were in the training set, while the other 4 patients were in the validation set. No significant differences between the two cohorts were noted regarding the clinico-pathological factors that we analyzed. The median follow-up time was 30 months and ranged from 15 to 100 months. The 5-year OS of the included patients was 68%. In total, 47 (10.4%) patients had lung metastases at the time of diagnosis, and the 5-year LMFS of the remaining patients was 62.4% (Figure 3).

**Table 1:** Characteristics of patients in the training and validation sets

Variable	Training Set	Validation Set	λ^2^	*P*-value
Gender				
Male	212	71	0.003	0.96
Female	128	43		
Age				
<14	103	44	2.69	0.10
≥14	237	70		
Histological type				
Osteoblastic	272	88	3.28	0.35
Chondroblastic	46	13		
Fibroblastic	10	5		
Others	12	8		
Enneking stage				
IIA	38	15	5.26	0.07
IIB	265	89		
III	37	10		
Tumor size			1.87	0.17
≤8 cm	156	40		
>8 cm	184	74		
Surgery type			0.87	0.35
Amputation	75	30		
Limb sparing	265	84		
WBC (mean ± SD) × 10^9^	8.1 ± 2.9	8.1 ± 3.2		0.96
Neutrophil (mean ± SD) × 10^9^	5.1 ± 2.3	4.9 ± 2.4		0.84
Lymphocyte (mean ± SD) × 10^9^	2.2 ± 0.7	2.2 ± 0.7		0.61
Platelet (mean ± SD) × 10^9^	294.6 ± 82.7	285.4 ± 83.6		0.31
Hemoglobin (mean ± SD) × 10^9^	130.8 ± 20.3	130.7 ± 19.1		0.95
Albumin (mean ± SD)	44.2 ± 5.2	43.3 ± 4.8		0.12
Fibrinogen (mean ± SD)	3.6 ± 1.3	3.7 ± 2.0		0.37
LDH (mean ± SD)	284.2 ± 294.2	251.8 ± 161.6		0.27
ALP (mean ± SD)	355.3 ± 634.1	292.8 ± 453.2		0.33

WBC: white blood cell; LDH: lactate dehydrogenase; ALP: alkaline phosphatase.

### Univariate and multivariate analysis of prognostic factors for OS and LMFS in the training set

The results of univariate analysis of the training set are presented in Table 2. The factors identified as potentially associated with 5-year OS included the pretreatment Enneking stage, tumor size, neutrophil count, PLT count, LDH level and ALP level (all *P* < 0.05). These potential factors were selected and analyzed using Cox proportional hazards models. The Enneking stage, tumor size, neutrophil count, PLT count, and ALP level maintained their prognostic significance for 5-year OS (all *P* < 0.05, Table 3). There was no significant difference in LDH level. The prognostic index of each risk factor for 5-year OS is summarized in Table 4A.

**Table 2:** Univariate analysis of clinical factors for 5-year overall survival (OS) and lung metastasis-free survival (LMFS) in the training set

Variable	Overall survival			LMFS		
	Patient	5-year OS	*P*-value	Patient	5-year LMFS	*P*-value
Gender						
Male	212	59.8	0.09	181	59.1	0.25
Female	128	69.7		122	62.4	
Age						
<14 years	103	58.0	0.72	91	56.8	0.25
≥14 years	237	56.0		212	62.0	
Histological type						
Osteoblastic	272	64.2	0.87	242	60.2	0.56
Chondroblastic	46	60.2		43	59.1	
Fibroblastic	10	70.1		8	56.0	
Others	12	55.3		10	60.4	
Enneking stage						
IIA	38	76.5	<0.01	38	79.4	0.05
IIB	265	59.5		265	58.2	
III	37	20.3		0		
Tumor size						
≤8 cm	156	80.9	<0.01	136	82.4	<0.01
>8 cm	184	41.4		167	43.5	
Surgery type						
Amputation	75	55.8	0.53	60	49.3	0.56
Limb sparing	265	61.6		243	54.7	
WBC × 10^9^						
<10	271	58.6	0.37	240	64.8	0.03
≥10	69	49.3		63	43.3	
Neutrophil × 10^9^						
<6.4	263	60.3	<0.01	234	65.1	0.004
≥6.4	77	42.0		69	43.5	
Lymphocyte × 10^9^						
<3.3	313	55.7	0.14	276	61.4	0.63
≥3.3	27	69.3		27	51.7	
Platelet × 10^9^						
<300	198	70.6	<0.01	182	76.4	<0.01
≥300	142	41.2		121	39.4	
Hemoglobin × 10^9^						
<130/120	206	51.0	0.40	189	60.2	0.92
≥130/120	134	62.2		114	60.1	
Albumin						
<35	8	75.0	0.14	7	85.7	0.41
35–50	311	55.1		277	59.8	
≥50	21	67.0		19	65.6	
Fibrinogen						
<4	268	58.4	0.15	240	65.6	0.03
≥4	72	51.1		63	43.3	
LDH						
<240	199	61.6	0.01	188	63.8	0.01
≥240	141	50.0		115	54.6	
ALP						
<110/150	103	88.8	<0.01	97	70.2	0.01
≥110/150	237	46.6		206	56.3	

WBC: white blood cell; LDH: lactate dehydrogenase; ALP: alkaline phosphatase; OS: overall survival; LMFS: lung metastasis-free survival.

**Table 3:** Multivariate analysis of clinical factors for 5-year overall survival (OS) and lung metastasis-free survival (LMFS) in the training set

Variable	5-year PS (*n* = 340)				5-year LMFS (*n* = 303)			
	β-value	RR	95% CI	*P*-value	β-value	RR	95% CI	*P*-value
Enneking stage	0.766	2.151	1.373–3.370	0.001	-	-	-	-
Tumor size	0.595	1.814	1.154–2.851	0.010	0.960	2.612	1.721–3.966	<0.001
Neutrophil × 10^9^	0.480	1.617	1.024–2.552	0.039	0.445	1.561	1.011–2.408	0.044
Platelet × 10^9^	0.482	1.619	1.060–2.473	0.026	0.467	1.595	1.091–2.330	0.016
ALP	1.421	4.140	1.907–8.987	< 0.001	0.551	1.736	1.084–2.779	0.022

OS: overall survival; LMFS: lung metastasis-free survival; ALP: alkaline phosphatase.

**Table 4A:** The Prognostic Index for 5-year Overall Survival

Risk factors	0	1	2	3	4
Enneking stage	IIA		IIB		III
Tumor size	≤8 cm	>8 cm			
Neutrophil × 10^9^	<6.4	≥6.4			
Platelet × 10^9^	<300	≥300			
ALP	<110/150			≥110/150	

The same procedure was performed for 5-year LMFS. Univariate analysis demonstrated that the Enneking stage, tumor size, WBC count, neutrophil count, PLT count, LDH level, and ALP level were significantly associated with 5-year PMFS (all *P* < 0.05, Table 2). Multivariate analysis revealed that only tumor size, neutrophil count, PLT count, and ALP level were independent prognostic factors for 5-year LMFS (all *P* < 0.05, Table 3). There were no significant differences in LDH level, WBC count or Enneking stage. The prognostic index of each risk factor for 5-year LMFS is summarized in Table 4B.

**Table 4B:** The Prognostic Index for 5-year lung metastasis free survival

Risk factors	0	1	2
Tumor size	≤8 cm		>8 cm
Neutrophil × 10^9^	<6.4	≥6.4	
Platelet × 10^9^	<300	≥300	
ALP	<110/150	≥110/150	

### Development of the prognostic index

**Figure 4:**
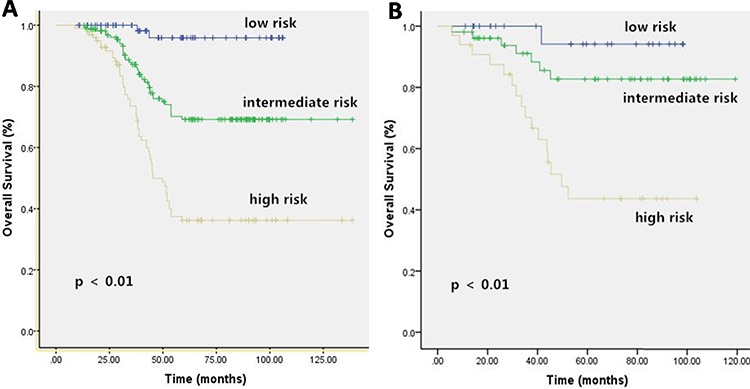
Kaplan-Meier Curves for Overall Survival (OS) in Training Set A. and Validation Set B **A.** Eleven subgroups of development cohort merged to form three categories with significantly different prognoses. **B.** There were significant statistical differences on 5 year OS among the three categories of validation cohort, according to our prognostic classification.

To calculate a prognostic index, each independent prognostic factor was assigned a number of points based on its regression coefficient. The sum of the points was regarded as the prognostic score for each patient. For 5-year OS, the prognostic index ranged from 0 to 10 points. Patients in the training set were then divided into 11 subgroups to perform survival estimates. Among these groups, three were identified as having significantly different prognoses (Figure 4A), as follows: patients with low risk (0–3 points), patients with intermediate risk (4–6 points) and patients with high risk (7–10 points).

**Figure 5:**
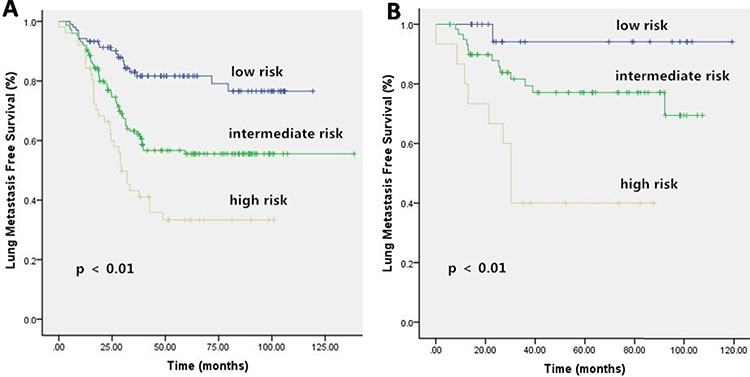
Kaplan-Meier Curves for Lung Metastasis Free Survival (LMFS) in Training Set A. and Validation Set B **A.** Five subgroups of development cohort merged to form three categories with significantly different prognoses. **B.** There were significant statistical differences on 5 year LMFS among the three categories of validation cohort, according to our prognostic classification.

For 5-year LMFS, the prognostic index ranged from 0 to 5 points using the same methods. Patients in the training set were divided into 6 subgroups to perform survival estimates. Three groups were identified that exhibited significantly different prognoses (Figure 5A): patients with low risk (0–1 points), patients with intermediate risk (2–3 points) and patients with high risk (4–5 points).

### Validation of the prognostic index

The validation set was analyzed based on the outcomes of the training set. After risk factors were identified in the training set, low-, medium- and high-risk groups were established. Then, the patients in the validation set were divided into 3 groups according to their prognostic indexes. The groups in the validation set and their prognostic outcomes are presented in Figures 4B and 5B. The C statistics for the 5-year OS and LMFS of the training and validation sets are summarized in Table 5 (all *P* < 0.05).

**Table 5:** C-statistic results

Items	C-statistic	95% confidence interval	*P*-value
5-year OS in the training set	0.723	0.676 to 0.771	<0.05
5-year LMFS in the training set	0.661	0.612 to 0.710	<0.05
5-year OS in the validation set	0.710	0.611 to 0.809	<0.05
5-year LMFS in the validation set	0.693	0.592 to 0.795	<0.05

OS: overall survival; LMFS: lung metastasis-free survival.

## DISCUSSION

Osteosarcoma is a heterogeneous cancer that exhibits wide variation in both clinical progression and prognosis. The Enneking staging system provides important clinical information for osteosarcoma, which is mainly diagnosed based on gross anatomy. However, the system has an obvious limitation as it ignores functional factors. Our study revealed that 5-year OS could be predicted by Enneking stage, tumor size, pretreatment neutrophil and PLT counts, and pretreatment ALP level. Moreover, tumor size, pretreatment neutrophil and PLT counts, and pretreatment ALP level were independent prognostic factors for 5-year LMFS. A pretreatment prognostic index derived by combining points for each of these features could accurately divide patients into low, intermediate and high risk groups for both death and lung metastasis.

Metastasis is the most crucial step for osteosarcoma treatment failure, and the lung is the most commonly involved organ [18]. Data regarding lung metastasis are continually collected and recorded at our institute. Therefore, we chose 5-year OS and LMFS as our endpoints to develop a multivariate Cox model and a prognostic index for lung metastasis and death. Additionally, pelvic and vertebral osteosarcomas are rare and associated with poor outcomes. It is improper to investigate the prognostic factors of these forms in conjunction with extremity osteosarcoma [19]. Therefore, our study focused on extremity osteosarcoma patients.

In general, Enneking stage and tumor size are definitive prognostic factors for osteosarcoma, as shown in Bielack's study [20]. A higher Enneking stage or larger tumor size may indicate greater malignancy and increased difficulty achieving adequate surgical margin. A cutoff of 8-cm maximal tumor diameter was demonstrated to predict death and lung metastasis in osteosarcoma, consistent with Kim's study [21].

To our knowledge, ALP level is considered a clinically useful marker of bone formation. Biochemical markers that reflect skeletal activity are thought to be sensitive indicators of early bone metabolism disturbances [22]. Bacci et al demonstrated that ALP level adversely affected 5-year event free survival in 789 patients with extremity osteosarcoma, a finding that is consistent with the results of our current study [23]. In a previous study of another patient cohort, we found that pretreatment ALP level predicted metastasis and poor prognosis in osteosarcoma patients [3]. Pretreatment ALP level remained prognostically significant for 5-year OS and LMFS in this cohort at our institute. The prognostic value of these 3 factors is consistent with 2 additional studies that developed 2 postoperative models to predict metastasis in Enneking stage IIB osteosarcoma [21, 24].

Interestingly, pretreatment PLT and neutrophil counts were predictive of death and lung metastases in the patients with extremity osteosarcoma. Based on preclinical data from other types of cancers [25], circulating PLTs might shield tumor cells from the host immune response [26], promote tumor cell migration and invasion [27], regulate angiogenesis, and maintain vascular integrity [28]. Takagi reported that PLTs could promote osteosarcoma cell growth by activating the PLT-derived growth factor receptor–Akt signaling axis [29]. Furthermore, pretreatment PLT and neutrophil counts are markers of host inflammation status. Higher pretreatment PLT and neutrophil counts may promote tumor cell metastasis and lead to poor outcomes [30, 31]. Lee et al demonstrated that a higher pretreatment neutrophil count adversely affected survival outcomes in cases of metastatic gastrointestinal stromal tumor treated with imatinib [32]. The definitive roles of PLTs and neutrophils and the underlying mechanisms should be accounted for in future studies and clinical trials. However, in contrast to the findings of a recent study of metastasis in osteosarcoma, age at diagnosis and gender were not associated with survival outcomes [33]. This may be due to the study of patients of different races or the use of different chemotherapy protocols.

To our knowledge, the C statistic is used to assess the predictive accuracy of a model. In the validation set, the C statistics for 5-year OS and LMFS were 0.710 and 0.693. These values were similar to those of Kim's study, which aimed to assess the 5-year probability of developing metastasis after neo-adjuvant chemotherapy and definitive surgery for AJCC stage II extremity osteosarcoma and found a C statistic of 0.78 [21]. A high C statistic indicates that the patients in the high-risk group are likely to have worse survival outcomes than those identified as having low risk according to the prognostic index. Therefore, our prognostic index was effective in predicting survival outcomes for extremity osteosarcoma.

The strengths of our study include the large number of osteosarcoma patients analyzed, the measurement of risk factors that can be easily obtained from routine tests, and their use to accurately classify patients into low-, intermediate-, and high-risk groups. Most importantly, this prognostic index for death and lung metastasis can be applied at the initiation of treatment. With respect to clinical trials, out prognostic index model could help study designers stratify risk, enroll comparable patients in different treatment arms and interpret trial outcomes. Second, the index could help clinicians apply individualized therapies according to the prognostic index at the time of diagnosis. For high-risk patients, doctors may advise closer monitoring, increased chemotherapy, or a more sensitive chemotherapy regimen. Third, this prognostic index may encourage researchers to investigate the mechanisms responsible for the higher risk of some patients, such as the molecular or genetic mechanisms. However, several limitations of our study also require consideration. First, only patients from one institute were included; this ensures treatment consistency but potentially limits the external validity of the findings. Second, it was a retrospective study. Third, the lack of functional assessment, such as the Musculoskeletal Tumor Society Score, and psychosocial outcomes was also a major limitation. Fourth, the included patients were all Mongolian which may make the results of this study less globally relevant. Finally, patients were enrolled from 2005 to 2013, which may increase the heterogeneity of the cohort. Therefore, this prognostic index should be prospectively validated at additional institutions.

## CONCLUSION

We developed a prognostic index to classify extremity osteosarcoma patients into low-, medium-, and high-risk groups for death and lung metastasis. These findings may be useful for clinicians performing risk stratification and designing individualized therapies at the time of diagnosis. These findings may also aid researchers in the design and interpretation of clinical trials.
